# Two-year trajectory of cognitive decline and neurological sequelae in COVID-19 survivors with acute neurological symptoms

**DOI:** 10.3389/fnagi.2026.1724803

**Published:** 2026-04-13

**Authors:** Jiashuo Lin, Shengcai Chen, Lei Zhang, Min Qiu, Wenbo Zuo, Luojinyun Wang, Quanwei He, Yanan Li, Huijuan Jin, Senwei Tan, Ming Huang, Canmin Zhu, Qiangjian Jin, Mengwen Wang, Yan Wan, Bo Hu

**Affiliations:** 1 Department of Neurology, Union Hospital, Tongji Medical College, Huazhong University of Science and Technology, Wuhan, China; 2 The Hubei Provincial Hospital of Integrated Chinese and Western Medicine, Wuhan, China; 3 The First People’s Hospital of Jiangxia District, Wuhan, China

**Keywords:** cognitive decline, COVID-19, neurological symptoms, post-acute sequelae, propensity score matching

## Abstract

**Introduction:**

The COVID-19 pandemic caused by SARS-CoV-2 has profound implications for global public health. It significantly affects the nervous system and cognitive function. The emergence of long COVID has raised concerns regarding long-term cognitive impairment, particularly among patients who experienced neurological symptoms during the acute phase of infection.

**Aim:**

This study investigated the impact of neurological symptoms during acute SARS-CoV-2 infection on subsequent cognitive change and neurological sequelae over a 2-year period.

**Methods:**

We enrolled 3,419 hospitalized patients with confirmed infection in Wuhan, China (Dec 2022–Mar 2023), and 2,087 completed follow-ups. Propensity score matching identified 901 patients with acute neurological symptoms and 901 controls. Cognitive decline was measured with the Informant Questionnaire on Cognitive Decline in the Elderly, and cognitive status with the Telephone Interview of Cognitive Status-40. Multivariable regression was used to examine risk factors and cognitive changes, and residual neurological symptoms and new-onset symptoms were also analyzed.

**Results:**

Acute neurological symptoms, particularly central nervous system manifestations such as delirium and brain fog, were strongly associated with both cognitive decline (aOR, 2.16; 95% CI, 1.53–3.07) and cognitive impairment (aOR, 2.75; 95% CI, 1.73–4.49). Delirium, brain fog, stroke, numbness, and facial paralysis were associated risk factors (all *p* < 0.05). After 2 years’ follow-up, most acute neurological symptoms had subsided, yet fatigue (8.66%) and brain fog (5.99%) persisted. Comorbidities did not significantly increase the risk of persistent symptoms. For the new-onset symptoms, the proportion of insomnia, tinnitus, blurred vision, movement disorder, palpitation, muscle weakness, and respiratory symptoms were much higher in the neurological symptom group (*p* < 0.05), with a highest proportion in insomnia (9.99% vs. 5.22%, *p* < 0.001), compared with the non-neurological symptom group.

**Conclusion:**

Acute neurological symptoms, especially central nervous system manifestations, were strongly associated with long-term cognitive decline and new-onset symptoms (mainly insomnia) in COVID-19 survivors. This study uniquely reveals the persistence of high levels in brain fog and fatigue at 2-year’s follow up, despite overall subsidence of most acute manifestations, underscoring the need for early intervention and sustained monitoring in this high-risk population.

## 1 Introduction

The COVID-19 pandemic caused by SARS-CoV-2 has profound implications for global public health. Recently, COVID-19 has caused approximately 780 million infections and over 7 million deaths (World Health Organization, 2025). Initially characterized by respiratory symptoms, its impact on multiple organ systems—especially the nervous system which was first reported by our team (Mao et al., 2020)—has become increasingly evident (Yang et al., 2021; Xie et al., 2022). These patients experienced neurological symptoms during the acute phase of infection and beyond, range from headache, dizziness, and anosmia to more severe conditions such as encephalitis and acute vascular events (Mao et al., 2020; Cho et al., 2023; Giussani et al., 2024).

Some symptoms that occur during the acute infection, along with newly emerged symptoms, may persist for a long time and have attracted a great deal of attention. Together, these were collectively referred to as post-acute sequelae of SARS-CoV-2 infection (PASC), also known as long COVID. Cognitive dysfunction—particularly manifestations like “brain fog” involving memory deficits, attention impairment, executive dysfunction, and slowed processing speed—is a predominant feature across diverse patient populations (McWhirter et al., 2023; Zhao et al., 2023). Upto 10.7–19.1% of COVID-19 survivors reported subjective cognitive decline after a 1–2-year follow-up (Liu et al., 2022; Araujo et al., 2025). Identified risk factors included age, severity of acute phase infection, dysregulation of glucose and lipid metabolism. The underlying mechanisms are thought to involve direct viral invasion of the central nervous system (CNS), systemic inflammation, and microvascular damage (Zubair et al., 2020). These findings suggest that neurological symptoms during acute SARS-CoV-2 infection may contribute to subsequent cognitive decline—particularly long-term impairment—and may serve as potential prognostic indicators. Although some studies have reported long-term follow-up results of COVID-19 patients (Giussani et al., 2024; Zhang et al., 2024), evidence remains limited regarding the specific COVID-19-related neurological manifestations and risk factors strongly associated with cognitive impairment. In addition, the long-term persistence of neurological symptoms following COVID-19, as well as the emergence of new-onset symptoms in COVID-19 survivors after recovery, warrant serious attention.

This study conducted a 2-year longitudinal follow-up of COVID-19 patients to explore the associations between acute-phase neurological symptoms and subsequent cognitive changes and neurological sequelae. By analyzing the epidemiological patterns of COVID-19-related neurological symptoms and their cognitive functional impacts, this research aims to identify key risk factors of COVID-19 infection associated with long-term neurological sequelae.

## 2 Materials and methods

### 2.1 Study population

This is a multicenter retrospective clinical study. Participants in this study were patients hospitalized with COVID-19 between December 2022 and March 2023 from nine hospitals in China, including the Wuhan Union Hospital, the Wuhan Union Hospital Chegu Campus, the Wuhan Union Hospital Jinyinhu Campus, the People’s Hospital of Dongxihu District, the Hubei Provincial Hospital of Integrated Chinese and Western Medicine, Hubei General Hospital, Wuhan Puren Hospital, The First People’s Hospital of Jiangxia District, Wuhan, and Wuhan Red Cross Hospital. Patients aged 18 years or older with firstly confirmed SARS-CoV-2 infection using reverse transcription-polymerase chain reactions or serological testing were included. Criteria for inclusion comprised: (1) identification of SARS-CoV-2 RNA in nasopharyngeal or oropharyngeal swabs through real-time reverse transcription polymerase chain reaction; and (2) consent to participate in the research. Exclusion criteria comprised: (1) inability to comprehend questionnaire items, or communication barriers due to linguistic or auditory limitations; (2) pre-existing self-reported or clinically diagnosed cognitive impairment; (3) first-degree familial history of dementia; (4) comorbid neurological conditions with potential cognitive impact; or (5) severe cardiovascular, hepatic, or renal dysfunction, or any malignant neoplasms.

### 2.2 Ethics approval

The study adhered to the ethical principles outlined in the 1975 Declaration of Helsinki and was approved by the Ethics Committee of Union Hospital, Tongji Medical College, Huazhong University of Science and Technology. Given that the study was conducted via telephone interviews, the requirement for written informed consent was waived. Instead, verbal informed consent was obtained from all participants or their legal guardians. Patient anonymity was strictly maintained throughout the study. This study followed the Strengthening the Reporting of Observational Studies in Epidemiology (STROBE) guidelines for cohort studies and was approved by the Ethics Committee of Wuhan Union Hospital.

### 2.3 Data collection

Trained physicians collected data on COVID-19—related factors for each participant from electronic hospital records—including nursing and medical notes, consultation reports, laboratory results, and imaging studies—or from a knowledgeable family member. Demographic characteristics, including age, sex, education level (defined by the number of years of education), and body mass index (BMI), were recorded. Comorbidities, such as hypertension (diagnosed according to the Joint National Committee on the Detection, Evaluation, and Treatment of High Blood Pressure guidelines), type 2 diabetes (diagnosed following the guidelines of the American Diabetes Association), hyperlipidemia (hypertriglyceridemia or hypercholesteremia), coronary heart disease (diagnosed according to the American College of Cardiology/American Heart Association guidelines), chronic obstructive pulmonary disease (COPD) (diagnosed following the Global Strategy for the Diagnosis, Management, and Prevention of COPD), liver disease (diagnosed according to the American Association for the Study of Liver Diseases guidelines), and chronic kidney disease [diagnosed following the Kidney Disease: Improving Global Outcomes (KDIGO) guidelines], were also documented. COVID-19-related clinical characteristics—including hospitalization duration, disease severity, ICU admission, and invasive ventilation—were assessed. COVID-19-related neurological symptoms at admission (delirium, brain fog, headache, dizziness, acute ischemic stroke, seizure, dysgeusia, anosmia, fatigue, myalgia, numbness, and facial paralysis) (Mao et al., 2020; Cho et al., 2023) were reviewed and confirmed by two neurologists. Among these, any occurrence of delirium, brain fog, headache, dizziness, or acute ischemic stroke was classified as indicating a CNS symptom (Mao et al., 2020; Cho et al., 2023). “Brain fog” is defined as a cluster of persistent subjective cognitive symptoms characterized primarily by impairments in attention, memory, and executive function, which may persist for months or even longer following illnesses and affect patients’ quality of life and daily functioning. “Brain fog” symptoms were reported by patients or their guardian, or documented in electronic medical records, and were subsequently evaluated and recorded by experienced neurologists, referring to previous researches (McWhirter et al., 2023; Hampshire et al., 2024; Denno et al., 2025). COVID-19 severity was classified according to the American Thoracic Society’s guidelines for community-acquired pneumonia, and severe COVID-19 was defined as the presence of either one major criterion or three or more minor criteria (Major criteria included septic shock requiring vasopressors and respiratory failure requiring mechanical ventilation; Minor criteria included a respiratory rate > 30 breaths/min, PaO2/FiO2 ratio < 250, multilobar infiltrates, etc.) (Metlay et al., 2019).

At 2-years follow-up after infection, patients were contacted via telephone to gather information on vaccine doses received, reinfection status, residual neurological symptoms and new-onset symptoms after SARS-CoV-2 infection (including Depression, Insomnia, Tinnitus, etc.), rehospitalization, and cognitive assessment outcomes. Current cognitive status was evaluated using the Chinese version of the TICS-40 (Fong et al., 2009), with a score of ≤ 20 indicating cognitive impairment. Cognitive decline over the previous 2 years was assessed using the Chinese version of the IQCODE scale (Fuh et al., 1995), with an IQCODE score ≥ 3.5 indicating longitudinal cognitive decline (sensitivity 92%, specificity 80%). The phone interviews were conducted by trained assessors (Jiashuo Lin, Min Qiu, Wenbo Zuo, and Mengwen Wang).

### 2.4 Statistical analysis

Continuous variables, including age and education level, were first tested for normality using the Shapiro-Wilk test and were found non-normally distributed. The Mann-Whitney U test was then used to compare the differences between two groups, and the results were presented as median and interquartile range (IQR). For categorical variables, such as gender, hypertension, and diabetes, etc., group differences were assessed using the Chi-square test, and the data were presented as frequencies and percentages. The study used a 1:1 propensity score matching (PSM) method to balance covariates that were considered potential influencers of cognitive outcomes. These covariates included demographic characteristics (age, gender, and education level), history of comorbidities (hypertension, diabetes, hyperlipidemia, coronary heart disease, COPD, liver disease, kidney disease), and COVID-19 infection-related factors, such as the number of infections, vaccination history, and whether the individual had severe COVID-19 during infection. Subsequently, the impact of neurological symptoms during COVID-19 infection on cognitive status at the 2-year follow-up was analyzed. Multivariate linear regression analyses examined the relationship between neurological symptoms and continuous cognitive outcomes (IQCODE or TICS-40 scores). Logistic regression was used to assess the associations between neurological symptoms and cognitive decline, and cognitive impairment. Covariates that were considered potential confounders of cognitive outcomes were adjusted for as comprehensively as possible, including demographic factors (e.g., age, sex, and education level), pre-existing medical conditions (e.g., liver disease, chronic kidney disease, hypertension), and COVID-19 infection-related factors, such as the number of infections, vaccination doses, and whether the individual had severe COVID-19 during infection. Results were reported as regression coefficients (β), standard errors (SE), *p*-values and OR (95% CI). Statistical significance was set at *p* < 0.05. All analyses were conducted using R software (version 4.3.3).

## 3 Results

### 3.1 Comparison of baseline characteristics before and after propensity score matching in patients with and without neurological symptoms during acute SARS-CoV-2 infection

**FIGURE 1:**
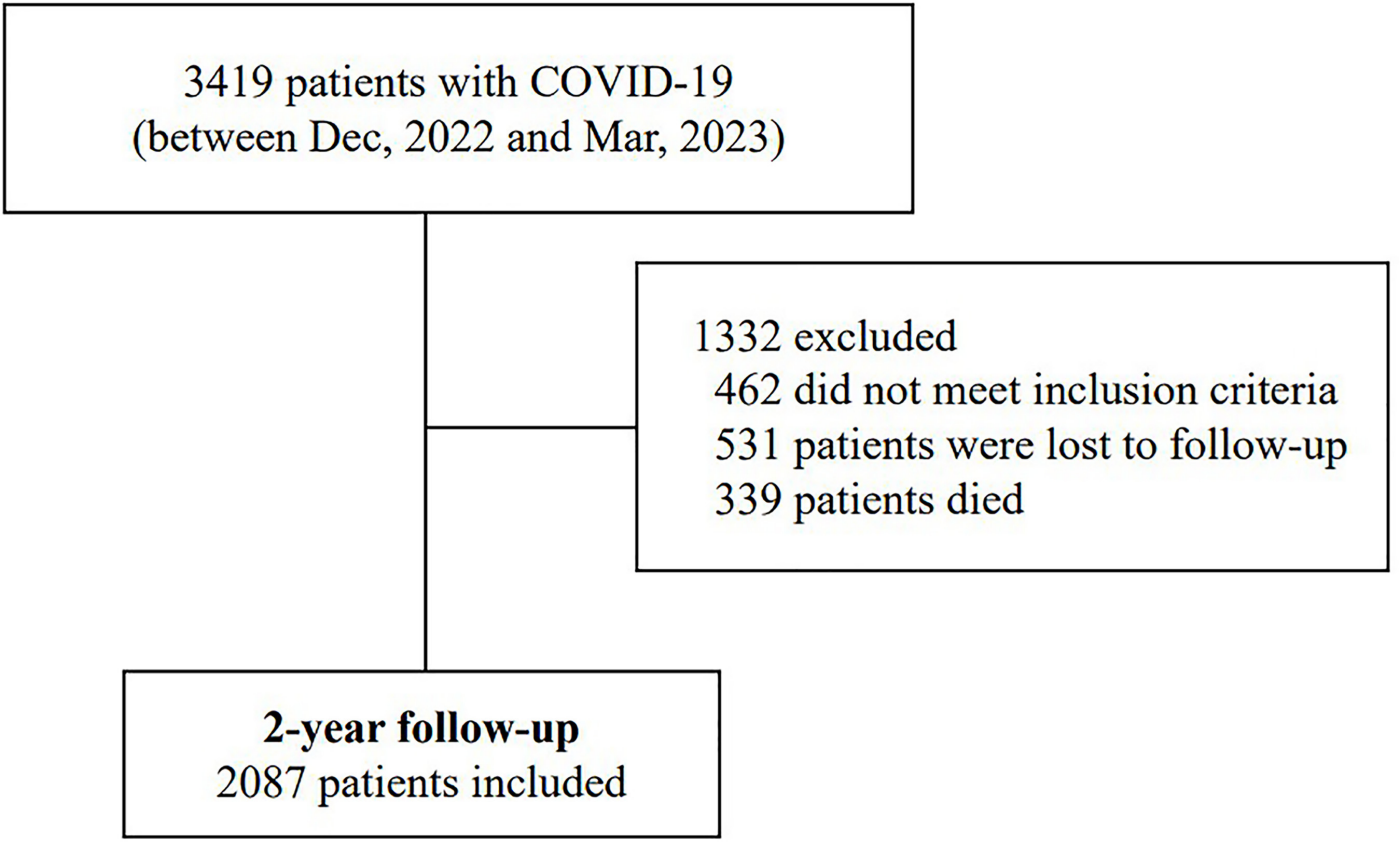
Flowchart of patient enrollment.

In this study, a total of 3,419 patients were screened, of whom 2,087 met the eligibility criteria and were included in the final analysis (Figure 1). Among them, 901 patients were assigned to the neurological symptoms group, and 1,186 to the non-neurological symptoms group. Then a PSM was conducted to balance covariates including demographic characteristics (age, gender, and education level), history of comorbidities (hypertension, diabetes, hyperlipidemia, coronary heart disease, COPD, liver disease, and kidney disease) (Austin, 2011). After PSM, both groups were adjusted to 901 patients each, resulting in a balanced distribution of these variables, with no significant differences between two groups (*p* > 0.05), enhancing comparability between the study and control groups (Table 1).

**TABLE 1:** Comparison of patient baseline data and post-PSM data.

Variable	Before PSM				After PSM			
	Total (*n* = 2,087)	COVID-19 without neurological symptom (*n* = 1,186)	COVID-19 with neurological symptom (*n* = 901)	*p*-value	Total (*n* = 1,802)	COVID-19 without neurological symptom (*n* = 901)	COVID-19 with neurological symptom (*n* = 901)	*p*-value
Age, median (Q1, Q3), y	63.00 (53.00, 71.00)	63.00 (54.00, 70.75)	63.00 (52.00, 71.00)	0.785	63.00 (53.00, 71.00)	63.00 (53.00, 70.00)	63.00 (52.00, 71.00)	0.667
Education, median (Q1, Q3), y	9.00 (6.00, 12.00)	9.00 (6.00, 12.00)	9.00 (6.00, 12.00)	0.006	9.00 (6.00, 12.00)	9.00 (6.00, 12.00)	9.00 (6.00, 12.00)	0.286
Male, *n* (%)	1,172 (56.16)	674 (56.83)	498 (55.27)	0.477	1,004 (55.72)	506 (56.16)	498 (55.27)	0.704
Hypertension, *n* (%)	863 (41.35)	475 (40.05)	388 (43.06)	0.166	775 (43.01)	387 (42.95)	388 (43.06)	0.962
Diabetes, *n* (%)	442 (21.18)	245 (20.66)	197 (21.86)	0.504	392 (21.75)	195 (21.64)	197 (21.86)	0.909
Hyperlipidemia, *n* (%)	402 (19.26)	214 (18.04)	188 (20.87)	0.105	367 (20.37)	179 (19.87)	188 (20.87)	0.599
Coronary heart disease, *n* (%)	231 (11.07)	126 (10.62)	105 (11.65)	0.458	209 (11.6)	104 (11.54)	105 (11.65)	0.941
COPD, *n* (%)	50 (2.4)	28 (2.36)	22 (2.44)	0.905	46 (2.55)	24 (2.66)	22 (2.44)	0.765
Liver disease, *n* (%)	277 (13.27)	184 (15.51)	93 (10.32)	< 0.001	178 (9.88)	85 (9.43)	93 (10.32)	0.528
Chronic kidney disease, *n* (%)	198 (9.49)	113 (9.53)	85 (9.43)	0.942	171 (9.49)	86 (9.54)	85 (9.43)	0.936

PSM, propensity score matching; COPD: chronic obstructive pulmonary disease.

### 3.2 Assessment of relationship between neurological symptoms and cognitive function

#### 3.2.1 Longitudinal cognitive decline in patients with neurological symptoms during acute SARS-CoV-2 infection

Longitudinal cognitive decline in patients was assessed by the IQCODE score and patients with acute neurological symptoms had significantly higher IQCODE scores than those without acute neurological symptoms [median (IQR), 3.125 (3.000, 3.385) vs. 3.000 (3.000, 3.250), *p* < 0.001]. In the multivariate linear regression analysis, a significant increase of the IQCODE score was still observed in patients with neurological symptoms after adjusting for confounding factors (age, gender, years of education, hypertension, etc.) (β = 0.068, *p* = 0.001) (Supplementary Table S1).

Further multivariate logistic regression analysis showed that age (aOR, 1.03; 95% CI, 1.02–1.04), chronic kidney disease (aOR, 1.66; 95% CI, 1.14–2.41), and infections numbers (aOR, 1.69; 95% CI, 1.32–2.17) were significant risk factors for cognitive decline (IQCODE ≥ 3.5), while years of education (aOR, 0.92; 95% CI, 0.90–0.95) served as a protective factor (Table 2). Additionally, patients with the neurological symptoms are more likely to experience cognitive decline than the control group (aOR, 1.38; 95% CI, 1.07–1.77). Moreover, within the subgroup exhibiting neurological symptoms, patients with CNS symptoms had a significantly higher risk of cognitive decline than those without CNS involvement (aOR, 2.16; 95% CI, 1.53–3.07) (Table 2).

**TABLE 2:** The multivariable logistic regression analysis related to IQCODE and TICS-40.

Variable	Multivariable logistic regression of cognitive decline (IQCODE ≥ 3.5)				Multivariable logistic regression of cognitive impairment (TICS-40 ≤ 20)			
	β	SE	*p*-value	aOR (95% CI)	β	SE	*p*-value	aOR (95% CI)
(Intercept)	−3.37	0.46	< 0.001	0.03 (0.01 ∼ 0.09)	−4.54	0.63	< 0.001	0.01 (0.00 ∼ 0.04)
Age	0.03	0.01	< 0.001	1.03 (1.02 ∼ 1.04)	0.04	0.01	< 0.001	1.04 (1.03 ∼ 1.06)
Male	0.11	0.13	0.384	1.12 (0.87 ∼ 1.44)	0.10	0.17	0.545	1.11 (0.80 ∼ 1.54)
Education	−0.08	0.02	< 0.001	0.92 (0.90 ∼ 0.95)	−0.09	0.02	< 0.001	0.91 (0.88 ∼ 0.95)
Hypertension	0.18	0.13	0.170	1.20 (0.92 ∼ 1.57)	0.29	0.17	0.093	1.34 (0.95 ∼ 1.88)
Diabetes	−0.18	0.15	0.234	0.83 (0.62 ∼ 1.12)	−0.12	0.19	0.546	0.89 (0.61 ∼ 1.30)
Hyperlipidemia	0.18	0.15	0.240	1.19 (0.89 ∼ 1.61)	0.42	0.19	0.023	1.53 (1.06 ∼ 2.20)
Coronary heart disease	−0.21	0.19	0.270	0.81 (0.55 ∼ 1.18)	−0.30	0.24	0.218	0.74 (0.46 ∼ 1.19)
COPD	−0.40	0.39	0.296	0.67 (0.31 ∼ 1.42)	−0.61	0.51	0.226	0.54 (0.20 ∼ 1.46)
Liver disease	−0.07	0.22	0.743	0.93 (0.61 ∼ 1.43)	−0.14	0.30	0.650	0.87 (0.49 ∼ 1.57)
Chronic kidney disease	0.51	0.19	0.008	1.66 (1.14 ∼ 2.41)	0.16	0.25	0.513	1.18 (0.72 ∼ 1.91)
Vaccination doses	−0.05	0.05	0.361	0.95 (0.86 ∼ 1.06)	−0.14	0.07	0.037	0.87 (0.77 ∼ 0.99)
Infections numbers	0.52	0.13	< 0.001	1.69 (1.32 ∼ 2.17)	0.22	0.17	0.188	1.24 (0.90 ∼ 1.72)
Severe COVID-19	0.28	0.17	0.094	1.32 (0.95 ∼ 1.83)	0.59	0.20	0.003	1.80 (1.22 ∼ 2.66)
Neurological symptoms	0.32	0.13	0.012	1.38 (1.07 ∼ 1.77)	0.31	0.17	0.063	1.36 (0.98 ∼ 1.89)
CNS-symptoms	0.77	0.18	< 0.001	2.16 (1.53∼3.07)	1.01	0.24	< 0.001	2.75 (1.73∼4.49)

aOR, adjusted odds ratio; SE, Standard Error; IQCODE, the Informant Questionnaire on Cognitive Decline in the Elderly; TICS-40, the Telephone Interview of Cognitive Status-40; COPD: Chronic Obstructive Pulmonary Disease; CNS: Central Nervous System.

#### 3.2.2 Current cognitive status of patients with neurological symptoms during acute SARS-CoV-2 infection

The current cognitive status of participants was determined by the TICS-40 score and there were no significant differences between patients with neurological symptoms and those without neurological symptoms [median (IQR): 25.00 (23, 29) vs. 25.00 (22, 28), *p* = 0.067]. Multivariate linear regression analysis of the TICS-40 scores still showed no significant correlation between the presence of neurological symptoms and the TICS-40 score (β = −0.415, *p* = 0.092), after adjusting for covariate (Supplementary Table S1).

In the multivariate logistic regression model for TICS-40 **≤** 20 (cognitive impairment), older age, hyperlipidemia, and severe COVID-19 were found significantly risk factors of cognitive impairment, while higher education level and a greater number of vaccination doses served as a protective factor against cognitive impairment (Table 2). It is noted that after adjusting for the confounding factors, patients with CNS symptoms had a significantly higher risk of current cognitive impairment (aOR, 2.75; 95% CI, 1.73–4.49) (Table 2).

### 3.3 Association between individual neurological symptoms and cognitive function

**FIGURE 2:**
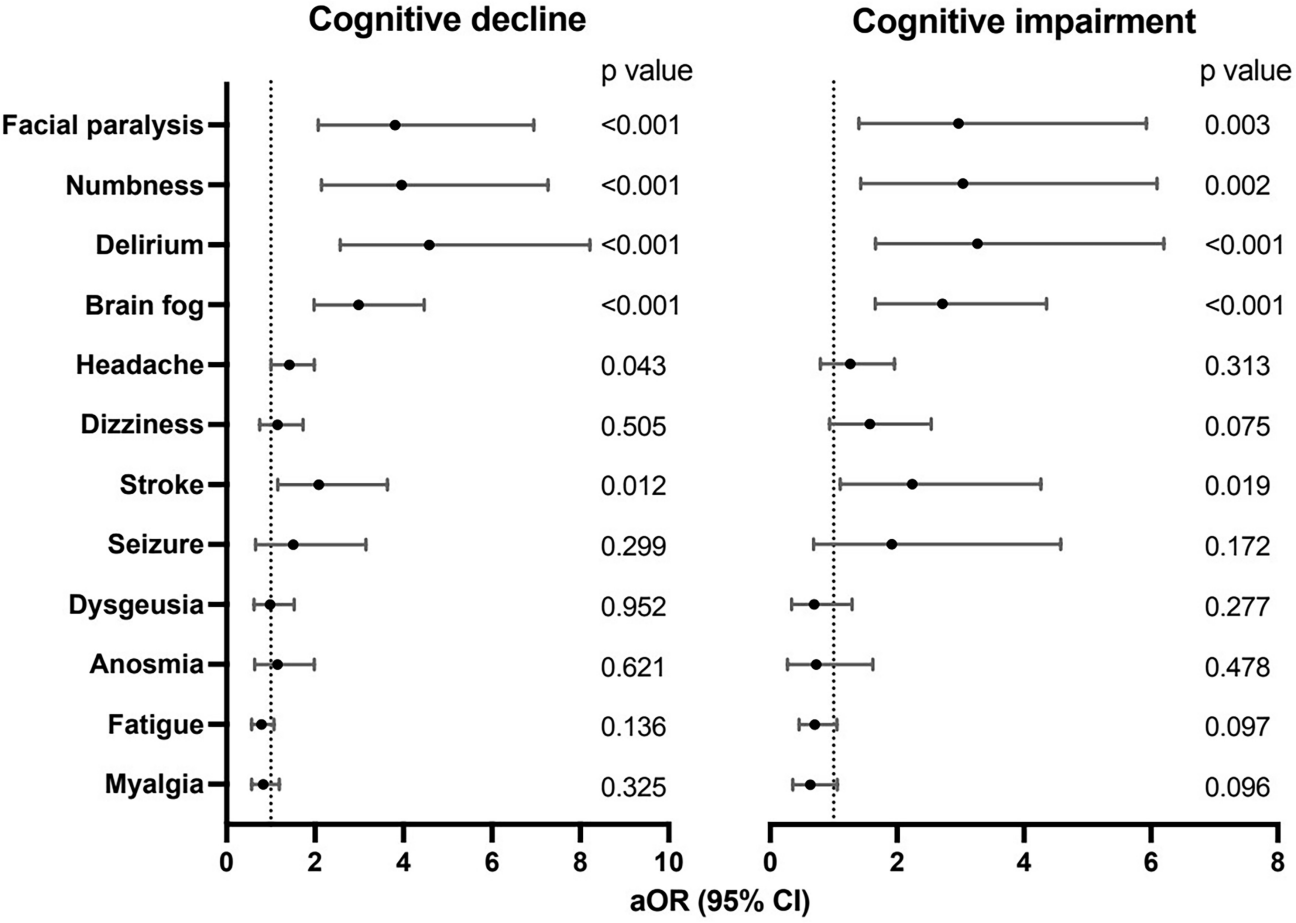
Associations of acute neurological symptoms with long-term cognitive decline and impairment.

Furthermore, we separately analyzed the association of each neurological symptom with cognitive outcomes using multivariate logistic regression. After adjusting for confounding factors such as demographic factors, pre-existing medical conditions, COVID-19 infection-related factors, etc., the results showed that delirium (aOR, 4.58; 95% CI, 2.57–8.22 for cognitive decline; aOR, 3.27; 95% CI, 1.66–6.20 for cognitive impairment), brain fog (aOR, 2.98; 95% CI, 1.98–4.47 for cognitive decline; aOR, 2.72; 95% CI, 1.66–4.36 for cognitive impairment), stroke (aOR, 2.08; 95% CI, 1.16–3.64 for cognitive decline; aOR, 2.24; 95% CI, 1.10–4.27 for cognitive impairment), numbness (aOR, 3.96; 95% CI, 2.15–7.27 for cognitive decline; aOR, 3.04; 95% CI, 1.43–6.10 for cognitive impairment), and facial paralysis (aOR, 3.81; 95% CI, 2.07–6.95 for cognitive decline; aOR, 2.97; 95% CI, 1.40–5.93 for cognitive impairment) were strong risk factors for both cognitive decline and cognitive impairment, while headache was associated with cognitive decline (aOR, 1.42; 95% CI, 1.00–1.99) rather than cognitive impairment (Table 3 and Figure 2).

**TABLE 3:** Multivariable logistic regression of single neurological symptoms and cognitive status.

Neurological symptoms	Multivariable logistic regression of cognitive decline (IQCODE ≥ 3.5) with different neurological symptoms			Multivariable logistic regression of cognitive impairment (TICS-40 ≤ 20) with different neurological symptoms		
	β	*p*-value	aOR	β	*p*-value	aOR
CNS-symptoms						
Delirium	1.52	< 0.001	4.58 (2.57 ∼ 8.22)	1.19	< 0.001	3.27 (1.66 ∼ 6.20)
Brain fog	1.09	< 0.001	2.98 (1.98 ∼ 4.47)	1.00	< 0.001	2.72 (1.66 ∼ 4.36)
Headache	0.35	0.043	1.42 (1.00 ∼ 1.99)	0.23	0.313	1.26 (0.79 ∼ 1.96)
Dizziness	0.14	0.505	1.15 (0.75 ∼ 1.73)	0.45	0.075	1.57 (0.94 ∼ 2.40)
Stroke	0.73	0.012	2.08 (1.16 ∼ 3.64)	0.81	0.019	2.24 (1.10 ∼ 4.27)
Seizure	0.41	0.299	1.51 (0.66 ∼ 3.15)	0.65	0.172	1.92 (0.69 ∼ 4.58)
PNS-symptoms						
Dysgeusia	−0.01	0.952	0.99 (0.62 ∼ 1.53)	−0.37	0.277	0.69 (0.34 ∼ 1.29)
Anosmia	0.14	0.621	1.15 (0.64 ∼ 1.99)	−0.32	0.478	0.73 (0.27 ∼ 1.62)
Fatigue	−0.24	0.136	0.79 (0.57 ∼ 1.07)	−0.36	0.097	0.70 (0.46 ∼ 1.05)
Myalgia	−0.19	0.325	0.83 (0.57 ∼ 1.19)	−0.46	0.096	0.63 (0.36 ∼ 1.06)
Numbness	1.38	< 0.001	3.96 (2.15 ∼ 7.27)	1.11	0.002	3.04 (1.43 ∼ 6.10)
Facial paralysis	1.34	< 0.001	3.81 (2.07 ∼ 6.95)	1.09	0.003	2.97 (1.4– ∼ 5.93)

aOR, adjusted odds ratio; IQCODE, the Informant Questionnaire on Cognitive Decline in the Elderly; TICS-40, the Telephone Interview of Cognitive Status-40; Central Nervous System; PNS: Peripheral Nervous System.

### 3.4 Distribution of the residual neurological symptoms in patients with acute SARS-CoV-2 infection

During the acute phase of SARS-CoV-2 infection, neurological symptoms were prevalent, and in this cohort, fatigue (39.96%), myalgia (28.86%), and headache (27.3%) have been the most commonly reported. In addition, symptoms such as brain fog (13.98%), dizziness (16.09%), and dysgeusia (16.98%) during COVID-19 infection also warrant attention. After 2 years follow-up, the proportions of most neurological symptoms were declined obviously, while fatigue (8.66%) and brain fog (5.99%) still maintained a high level in these patients (Table 4).

**TABLE 4:** Neurological symptoms during acute COVID-19 infection and residual neurological symptoms after recovery.

Neurological symptoms	Neurological symptoms during COVID-19 infection and residual symptoms at the 2-year follow-up	
	Neurological symptoms during COVID-19 infection	Residual neurological symptoms at the 2-year follow-up
CNSg		
Delirium (*n*, %)	56 (6.22)	0
Brain fog (*n*, %)	126 (13.98)	54 (5.99)
Headache (*n*, %)	246 (27.30)	23 (2.55)
Dizziness (*n*, %)	145 (16.09)	8 (0.89)
Stroke (*n*, %)	61 (6.77)	0
Seizure (*n*, %)	44 (4.88)	11 (1.22)
PNS		
Dysgeusia (*n*, %)	153 (16.98)	14 (1.55)
Anosmia (*n*, %)	84 (9.32)	12 (1.44)
Fatigue (*n*, %)	360 (39.96)	78 (8.66)
Myalgia (*n*, %)	260 (28.86)	15 (1.66)
Numbness (*n*, %)	52 (5.77)	3 (0.33)
Facial paralysis (*n*, %)	53 (5.88)	0

CNS, central nervous system; PNS, peripheral nervous system.

Within the subgroup of patients who developed neurological symptoms during acute COVID-19, we further compared those who retained at least one neurological symptom at follow-up (i.e., neurological symptoms that emerged during acute COVID-19 and persisted) with those who had no residual neurological sequelae (i.e., all neurological symptoms that arose during acute COVID-19 had completely resolved) in Supplementary Table S2. A higher proportion of females was observed in the group with residual symptoms (52.75% vs. 47.25%, *p* = 0.015). In addition, the distribution of vaccination doses also differed significantly between the two groups (*p* = 0.023). No statistically significant differences were found with respect to medical history (all *p* > 0.05), suggesting no effect on the persistence of post-COVID neurological symptoms.

### 3.5 New-onset symptoms of SARS-CoV-2 infection and their associations with neurological symptoms

Apart from the residual neurological symptoms, new-onset sequelae of SARS-CoV-2 infection during the follow-up period were also recorded and compared between the two groups. Among all the new-onset symptoms, depression (7.05%), insomnia (7.60%) and stroke (9.32%) were mostly common, while other symptoms are rare, with a proportion of less than 5% (Table 5). Compared with the non-neurological symptom group, the neurological symptom group exhibited a significantly higher incidence of total new-onset symptoms (40.84% vs. 21.42%, *p* < 0.001). And the proportion of symptoms like insomnia, tinnitus, blurred vision, movement disorder, Palpitation, muscle weakness, and respiratory symptoms were much higher in the neurological symptom group (*p* < 0.05), with a high proportion in insomnia (9.99%).

**TABLE 5:** Distribution of new-onset symptoms after SARS-CoV-2 infection.

New-onset symptoms after recover of COVID-19	Total (*n* = 1,802)	COVID-19 without neurological symptom (*n* = 901)	COVID-19 with neurological symptom (*n* = 901)	*p*-value
Total new-onset symptoms (*n*, %)	561 (31.13)	193 (21.42)	368 (40.84)	< 0.001
Depression (*n*, %)	127 (7.05)	57 (6.33)	70 (7.77)	0.232
Insomnia (*n*, %)	137 (7.60)	47 (5.22)	90 (9.99)	< 0.001
Tinnitus (*n*, %)	20 (1.11)	4 (0.44)	16 (1.78)	0.007
Blurred vision (*n*, %)	39 (2.16)	6 (0.67)	33 (3.66)	< 0.001
Dysphagia (*n*, %)	9 (0.50)	3 (0.33)	6 (0.67)	0.504
Non-chest pain (*n*, %)	12 (0.67)	6 (0.67)	6 (0.67)	1.000
Autonomic neuropathy (*n*, %)	33 (1.83)	13 (1.44)	20 (2.22)	0.219
Movement disorder (*n*, %)	87 (4.83)	32 (3.55)	55 (6.10)	0.011
Sensitive disorder (*n*, %)	79 (4.38)	36 (4.00)	43 (4.77)	0.421
Palpitation (*n*, %)	25 (1.39)	5 (0.55)	20 (2.22)	0.003
Chest-pain (*n*, %)	9 (0.50)	4 (0.44)	5 (0.55)	1.000
Appetite loss (*n*, %)	39 (2.16)	17 (1.89)	22 (2.44)	0.418
Muscle weakness (*n*, %)	52 (2.89)	12 (1.33)	40 (4.44)	< 0.001
Hair loss (*n*, %)	13 (0.72)	3 (0.33)	10 (1.11)	0.051
Gastrointestinal symptoms (*n*, %)	17 (0.94)	6 (0.67)	11 (1.22)	0.223
Respiratory symptoms (*n*, %)	88 (4.88)	15 (1.66)	73 (8.10)	< 0.001
Rash (*n*, %)	12 (0.67)	5 (0.55)	7 (0.78)	0.562
Stroke (*n*, %)	168 (9.32)	73 (8.10)	95 (10.54)	0.075

For new-onset symptoms with an incidence greater than 5%—namely depression, insomnia, and stroke—we conducted further subgroup analyses in Supplementary Table S3. For new-onset stroke, significant differences were observed in age, sex, education level, and history of hypertension, hyperlipidemia and liver disease (*p* < 0.05). For new-onset depression, significant differences were observed in education level and hyperlipidemia history (*p* < 0.05). For new-onset insomnia, significant differences were found in age, sex, and the number of COVID-19 vaccine doses received (*p* < 0.05).

## 4 Discussion

Although previous studies have described the prevalence of cognitive impairment following COVID-19, our study systematically evaluated a broad spectrum of acute neurological manifestations—including both central and peripheral nervous system symptoms. Their associations with cognitive decline and impairment were quantified over a 2-year period, with propensity score matching applied to minimize potential confounding. It was demonstrated that acute CNS manifestations (e.g., delirium and brain fog) remained independent risk factors for cognitive decline (aOR 2.16) and cognitive impairment (aOR 2.75), even after adjustment for comorbidities. Furthermore, although the majority of acute symptoms resolved over time, relatively high residual rates of fatigue (8.66%) and brain fog (5.99%) were observed at 2-years’ follow-up. Although most new-onset symptoms after SARS-CoV-2 infection were relatively rare (< 5%), the proportion of insomnia, tinnitus, blurred vision, movement disorder, palpitation, muscle weakness, and respiratory symptoms were much higher in the neurological symptom group, with a highest proportion in insomnia (9.99%), compared with the non-neurological symptom group. Collectively, these results underscore the prognostic value of acute CNS symptoms for targeted intervention strategies and address a critical gap in evidence regarding the need for sustained long-term monitoring of COVID-19 patients with neurological involvement.

Our findings add to the growing evidence that SARS-CoV-2 infection is a significant risk factor for cognitive impairment. A previous study reported that the prevalence of cognitive impairment was 19.1% among hospitalized COVID-19 patients—nearly twice that of non-hospitalized patients (10.7%) and significantly higher than that observed in uninfected controls (3.2%) (Araujo et al., 2025). Community-based cohorts also revealed that individuals hospitalized with COVID-19 demonstrated poorer performance in executive function and memory compared with non-hospitalized counterparts (Demmer et al., 2025). Longitudinal analyses from the UK PROTECT study further indicated that COVID-19 infection was identified as one of the major contributing factors to accelerate cognitive decline in older adults during the pandemic (Corbett et al., 2023). Among patients with long COVID, 27% exhibited global cognitive impairment, with particularly pronounced deficits in attention, working memory, processing speed, and verbal fluency (Serrano Del Pueblo et al., 2024).

Neurological symptoms during acute SARS-CoV-2 infection serve as potential prognostic indicators of subsequent cognitive decline—particularly long-term impairment. For example, previous studies have shown that delirium during hospitalization for COVID-19 was associated with functional impairment and cognitive decline within 6 months after discharge (OR, 2.48; 95% CI, 1.38–4.82) (Kaushik et al., 2024). One cross-sectional study reported that patients who experienced neurological symptoms such as headache, anosmia, or dysgeusia during infection performed worse on cognitive testing 10–34 days after discharge (Almeria et al., 2023). However, these studies did not specifically examine the association between multiple neurological symptoms and long-term cognition. Our study not only assessed the overall relationship between the presence of neurological symptoms and cognitive outcomes but also further disentangled the impact of individual symptoms on long-term cognitive trajectories. Also, individual neurological symptoms during acute infection—including delirium, brain fog, headache, stroke, numbness, and facial paralysis—were all identified as risk factors for cognitive decline at 2 years post-infection, may be linked to that SARS-CoV-2 has already exerted neuroinvasive effects. This suggests that the occurrence of these symptoms during acute COVID-19 infection warrants particular attention from neurologists, with timely cognitive assessments to enable early detection of cognitive changes and to guide appropriate intervention strategies.

It is important that in our findings, the CNS-symptoms—brain fog—were not only significantly associated with cognitive decline and cognitive impairment at 2-years’ follow-up, but also still maintained at a high level. Characterized by ongoing subjective cognitive complaints, brain fog can persist long after the initial illness has resolved, frequently impairing patients’ daily functioning and overall wellbeing (Hampshire et al., 2024; Qin et al., 2024). Its underlying mechanisms may be linked to persistent neuroinflammation, immune system activation, and disruption of the blood-brain barrier following COVID-19 infection, all of which can accelerate cognitive deterioration (Greene et al., 2024).

Systematic reviews reported that neurological symptoms are common sequelae of COVID-19, often persisting over extended trajectories and exerting substantial impact on quality of life (Giussani et al., 2024). A prospective cohort study from Germany reported that neurological symptoms alone accounted for as much as 61.5% of long COVID sequelae (Bahmer et al., 2022). Another bidirectional, longitudinal cohort study demonstrated that 55% of patients continued to exhibit at least one sequela at 2-year follow-up (Huang et al., 2022), a prevalence inconsistent with our findings. However, in our cohort, the proportion of sequelae was much lower at 2-year follow-up. This discrepancy likely stems from differences in viral pathogenicity. Our study focused exclusively on Omicron cases (December 2022–March 2023), contrasting with UK findings on the original strain, suggesting that Omicron is associated with a lower risk of long-term sequelae.

It is interesting that some patients who experienced neurological symptoms during acute SARS-CoV-2 infection continued to exhibit sequelae throughout the 2-year follow-up period, warranting further attention. Then, a further comparative analysis between patients with persistent symptoms and those without revealed that a higher proportion of women and fewer vaccinations are the risk factors rather than comorbidities. This is consistent with a previous case-control study comparing long COVID patients with predominant neurological symptoms to fully recovered individuals, in which a markedly higher proportion of women in the long COVID group (71% vs. 36%) was reported (Serrano Del Pueblo et al., 2024). The overrepresentation of women in the persistent-symptom group raises the possibility of sex-specific biological mechanisms, such as differences in immune responses, hormonal regulation, or susceptibility to neuroinflammatory processes following viral infection. The protective effect observed with higher vaccination doses may reflect reduced viral persistence or attenuation of immune-mediated damage. Alternatively, vaccination could mitigate the severity of acute infection, thereby lowering the risk of subsequent neurological sequelae. These observations underscore the importance of close monitoring in vulnerable subgroups and highlight the need for further research into underlying mechanisms, as well as validation in larger and more diverse cohorts.

Moreover, new-onset symptoms following SARS-CoV-2 infection is a key point worthy of particular attention. A previous Chinese cohort study of 125 patients reported that 26.45% and 9.92% developed insomnia and depressive symptoms, respectively, shortly after recovery, but it is limited by small sample size and assessing only immediate post-recovery outcomes (Xu et al., 2022). In our study, we found patients with acute-phase neurological symptoms were significantly more likely to develop new neurological symptoms at 2-year follow-up (40.84%), and depression, insomnia, and stroke are three most common newly developed neuropsychiatric symptoms. In addition, our findings revealed distinct risk profiles for new-onset sequelae: (1) patients with new stroke were generally older, predominantly male, and frequently had comorbid hypertension and hyperlipidemia, consistent with traditional vascular risk factors; (2) new-onset depression was more common among patients with hyperlipidemia and lower educational attainment, suggesting an interplay of metabolic and psychosocial determinants; (3) new-onset insomnia was more frequent among older patients, women, and those with fewer vaccine doses, implying that demographic characteristics and immunization status may influence susceptibility to sleep disturbances. These results highlight the diverse manifestations of long COVID and necessitate symptom-specific risk stratification. Future research should prioritize elucidating the mechanisms underlying these multi-system interactions.

Nonetheless, our study has several limitations. First, the epidemiological context during our enrollment period (December 2022–March 2023) coincided with a nationwide Omicron wave in China, during which universal testing policies revealed exceptionally high infection rates, leaving few truly uninfected individuals available for recruitment as controls. Consequently, cognitive changes in healthy individuals over the same 2-year period could not be directly assessed and could only be inferred from previous cohort studies. Second, the IQCODE relies on retrospective informant reports, which may introduce recall bias. Relatives’ or caregivers’ judgments of cognitive decline could be influenced by their emotional state, illness perceptions, or media exposure. Thus, the inherent subjectivity of this instrument should be considered when interpreting our findings. Third, despite the use of propensity score matching, multivariate logistic regression and subgroup analysis to control confounding factors, residual confounding from unmeasured variables (e.g., genetic susceptibility and socioeconomic status) may persist and potentially bias our estimates. Forth, our study focused on hospitalized Omicron patients during a specific epidemic wave. The generalizability of our findings to pre-Omicron variants or other geographic regions and healthcare systems requires further investigation through larger, multicenter studies with diverse populations and viral variants. Finally, given the close relationship between cognition and aging, extended follow-up over the next 5–10 years is essential to fully capture long-term outcomes.

## 5 Conclusion

Acute neurological symptoms, particularly CNS symptoms, like delirium and brain fog, are significantly associated with long-term cognitive decline in COVID-19 survivors. Patients with these symptoms are at higher risk for either ongoing cognitive impairment or decline over time and more likely to have a higher incidence of new-onset symptoms at the 2-year follow-up, highlighting the importance of early intervention and long-term monitoring in this subgroup.

## Data Availability

The original contributions presented in the study are included in the article/Supplementary material, further inquiries can be directed to the corresponding authors.
