# Supplementary material for: Two-year trajectory of cognitive decline and neurological sequelae in COVID-19 survivors with acute neurological symptoms
Source: Front Aging Neurosci. 2026 Apr 13;18:1724803. doi: 10.3389/fnagi.2026.1724803 (PMC13111338; doi:10.3389/fnagi.2026.1724803)
Supplement: Supplementary file 1 [file Data_Sheet_1.docx]

**Supplementary Table 1. Multivariable Linear Regression for IQCODE and TICS-40**

|  | **Multivariate Linear Regression of IQCODE Scores** | | |  | **Multivariate Linear Regression of TICS-40 Scores** | | |
| --- | --- | --- | --- | --- | --- | --- | --- |
| **Variable** | **β** | **SE** | ***p* Value** |  | **β** | **SE** | ***p* Value** |
| (Intercept) | 2.980 | 0.069 | <0.001 |  | 29.899 | 0.855 | <0.001 |
| Age | 0.005 | 0.001 | <0.001 |  | -0.105 | 0.010 | <0.001 |
| Male | 0.014 | 0.020 | 0.486 |  | -0.238 | 0.249 | 0.339 |
| Education | -0.012 | 0.002 | <0.001 |  | 0.187 | 0.029 | <0.001 |
| Hypertension | 0.018 | 0.022 | 0.417 |  | -0.446 | 0.269 | 0.098 |
| Diabetes | -0.023 | 0.024 | 0.345 |  | 0.397 | 0.302 | 0.189 |
| Hyperlipidemia | 0.024 | 0.024 | 0.330 |  | -0.515 | 0.301 | 0.087 |
| Coronary heart disease | -0.034 | 0.031 | 0.271 |  | 0.311 | 0.389 | 0.424 |
| COPD | -0.054 | 0.062 | 0.384 |  | 1.341 | 0.775 | 0.084 |
| Liver Disease | -0.040 | 0.033 | 0.222 |  | 0.220 | 0.406 | 0.587 |
| Chronic kidney disease | 0.077 | 0.034 | 0.023 |  | -0.510 | 0.421 | 0.226 |
| Vaccination doses | -0.009 | 0.009 | 0.297 |  | 0.267 | 0.109 | 0.014 |
| Infections numbers | 0.063 | 0.023 | 0.005 |  | -0.185 | 0.281 | 0.511 |
| Severe COVID-19 | 0.139 | 0.028 | <0.001 |  | -1.692 | 0.349 | <0.001 |
| Neurological symptoms | 0.068 | 0.020 | 0.001 |  | -0.415 | 0.246 | 0.092 |

OR, odds ratio; SE, Standard Error；TICS-40, the Telephone Interview of Cognitive Status-40;

**Supplementary Table 2. Group differences between patients with and without residual neurological symptoms**

| **Variable** | **Total (n=901)** | **Without residual neurological symptoms (n=719)** | **With residual neurological symptoms (n=182)** | **p** |
| --- | --- | --- | --- | --- |
| Age, M (Q₁, Q₃) | 63.00 (52.00, 71.00) | 63.00 (52.00, 71.00) | 63.00 (54.00, 70.00) | 0.557 |
| Education, M (Q₁, Q₃) | 9.00 (6.00, 12.00) | 9.00 (6.00, 12.00) | 9.00 (6.00, 16.00) | 0.058 |
| Male, n (%) | 498 (55.27) | 412 (57.30) | 86 (47.25) | 0.015 |
| Hypertension, n (%) | 388 (43.06) | 312 (43.39) | 76 (41.76) | 0.691 |
| Diabetes, n (%) | 197 (21.86) | 156 (21.70) | 41 (22.53) | 0.809 |
| Hyperlipidemia, n (%) | 188 (20.87) | 143 (19.89) | 45 (24.73) | 0.151 |
| Coronary heart disease, n (%) | 105 (11.65) | 87 (12.10) | 18 (9.89) | 0.407 |
| COPD, n (%) | 22 (2.44) | 17 (2.36) | 5 (2.75) | 0.976 |
| Liver Disease, n (%) | 93 (10.32) | 77 (10.71) | 16 (8.79) | 0.447 |
| Chronic kidney disease, n (%) | 85 (9.43) | 65 (9.04) | 20 (10.99) | 0.422 |
| Vaccination doses, M (Q₁, Q₃) | 3.00 (2.00, 3.00) | 3.00 (2.00, 3.00) | 3.00 (2.00, 3.00) | 0.023 |
| Infections numbers, M (Q₁, Q₃) | 1.00 (1.00, 1.00) | 1.00 (1.00, 1.00) | 1.00 (1.00, 1.00) | 0.235 |
| Severe COVID-19, n (%) | 177 (19.64) | 137 (19.05) | 40 (21.98) | 0.375 |

**Supplementary 3. Subgroup analysis of risk factors associated with new-onset stroke, depression, and insomnia among post-acute COVID-19 patients**

|  | **New-onset Stroke** | | |  | **New-onset Depression** | | |  | **New-onset Insomnia** | | |
| --- | --- | --- | --- | --- | --- | --- | --- | --- | --- | --- | --- |
| **Variable** | **Without new onset stroke** | **With new onset stroke** | **p** |  | **Without new onset depression** | **With new onset depression** | **p** |  | **Without new onset insomnia** | **With new onset insomnia** | **p** |
| **Age, M (Q₁, Q₃)** | 62 (52, 70) | 68 (61.8, 75) | <0.001 |  | 63 (53, 71) | 63 (54, 70) | 0.8659 |  | 63 (52, 71) | 65 (57, 72) | 0.0406 |
| **Male, n (%)** | 888 (54.3) | 116 (69) | 0.0004 |  | 944 (56.4) | 60 (47.2) | 0.0573 |  | 940 (56.5%) | 64 (46.7%) | 0.0343 |
| **Education, M (Q₁, Q₃)** | 9 (6, 12) | 9 (6, 12) | 0.0494 |  | 9 (6, 12) | 9 (6, 12) | 0.0174 |  | 9 (6, 12) | 9 (6, 12) | 0.172 |
| **Hypertension, n (%)** | 672 (41.1) | 103 (61.3) | <0.001 |  | 731 (43.6) | 44 (34.6) | 0.0599 |  | 712 (42.8) | 63 (46) | 0.5205 |
| **Diabetes, n (%)** | 346 (21.2) | 46 (27.4) | 0.0787 |  | 363 (21.7) | 29 (22.8) | 0.8456 |  | 358 (21.5) | 34 (24.8) | 0.4257 |
| **Hyperlipidemia, n (%)** | 318 (19.5) | 49 (29.2) | 0.0041 |  | 325 (19.4) | 42 (33.1) | 0.0004 |  | 340 (20.4) | 27 (19.7) | 0.9293 |
| **Coronary heart disease, n (%)** | 182 (11.1) | 27 (16.1) | 0.0759 |  | 195 (11.6) | 14 (11) | 0.9473 |  | 189 (11.4) | 20 (14.6) | 0.3163 |
| **COPD, n (%)** | 41 (2.5) | 5 (3) | 0.9135 |  | 43 (2.6) | 3 (2.4) | 1.000 |  | 41 (2.5) | 5 (3.6) | 0.572 |
| **Liver disease, n (%)** | 173 (10.6) | 5 (3) | 0.0026 |  | 169 (10.1) | 9 (7.1) | 0.3476 |  | 166 (10) | 12 (8.8) | 0.7584 |
| **Chronic kidney disease, n (%)** | 156 (9.5) | 15 (8.9) | 0.9027 |  | 162 (9.7) | 9 (7.1) | 0.4229 |  | 158 (9.5) | 13 (9.5) | 1.000 |
| **Vaccination doses, n (%)** | 3 (2, 3) | 3 (2, 3) | 0.6849 |  | 3 (2, 3) | 3 (2, 3) | 0.990 |  | 3 (2, 3) | 3 (2, 3) | 0.0405 |
| **Infections numbers, n (%)** | 1 (1, 1) | 1 (1, 1) | 0.257 |  | 1 (1, 1) | 1 (1, 1) | 0.1194 |  | 1 (1, 1) | 1 (1, 1) | 0.0882 |
| **Severe COVID-19, n (%)** | 233 (14.3) | 26 (15.5) | 0.7546 |  | 243 (14.5) | 16 (12.6) | 0.6455 |  | 237 (14.2) | 22 (16.1) | 0.6467 |
